# Extracellular Vesicles and Their Role in Skin Inflammatory Diseases: From Pathogenesis to Therapy

**DOI:** 10.3390/ijms26083827

**Published:** 2025-04-18

**Authors:** Xuan Lei, Sabine Ring, Shiying Jin, Sonali Singh, Karsten Mahnke

**Affiliations:** Department of Dermatology, University Hospital Heidelberg, Im Neuenheimer Feld 440, 69120 Heidelberg, Germany; xuan.lei@med.uni-heidelberg.de (X.L.); sabine.ring@med.uni-heidelberg.de (S.R.); shiying.jin@med.uni-heidelberg.de (S.J.); sonali.singh@med.uni-heidelberg.de (S.S.)

**Keywords:** extracellular vesicles, psoriasis, atopic dermatitis, systemic lupus erythematosus, wound healing

## Abstract

Extracellular vesicles (EVs), including exosomes, microvesicles, and apoptotic bodies, are released into the extracellular space by almost all known cell types. They facilitate communication between cells by transferring bioactive molecules, which impact both physiological processes and the development of diseases. EVs play a crucial role in the pathogenesis of various diseases by participating in multiple pathological processes. They contribute to disease progression by triggering cytokine release, modulating immune cell activity, and inducing inflammatory and immune responses. Beyond their pathological implications, EVs also offer significant therapeutic potential. Both natural and engineered EVs show great potential in the fields of targeted therapy, drug delivery, and immune modulation in dermatological applications. The development of EV-based treatments is showing promise in advancing patient outcomes, particularly in chronic inflammatory and immune-mediated skin conditions. This review comprehensively examined the biogenesis, classification, and functional roles of EVs, including advanced methods for their isolation and characterization. Furthermore, we summarized recent studies highlighting the involvement of EVs in four major inflammatory skin diseases: psoriasis, atopic dermatitis, systemic lupus erythematosus, and wound healing.

## 1. Introduction

Extracellular vesicles (EVs) are membranous structures originating from the endosomal system or the plasma membrane of cells. EVs are released by nearly all healthy and pathologically altered eukaryotic cells and can be detected in various biological fluids such as blood, urine, saliva, and breast milk. They are vital for facilitating communication between cells by delivering bioactive molecules, including proteins, lipids, and nucleic acids (such as RNA and DNA), thus influencing the functions of recipient cells. Depending on their size and biogenesis, EVs are categorized into exosomes, microvesicles, and apoptotic bodies.

The study of EVs dates back to the 20th century. In 1967, Peter Wolf first observed that platelets release granule-derived molecules that promote coagulation. The term “platelet dust” was used at the time to describe this subcellular coagulant material [1]. Later, in 1970, Webber and Johnson observed that activated platelets release vesicles containing materials from platelet alpha granules, which contribute to blood clotting [2]. In 1971, Crawford further investigated platelet-derived microparticles, confirming that microparticles in pig plasma contained ATPase activity, contractile proteins, and lipids [3]. Subsequently, R.M. Johnstone described the release of membrane vesicles during the differentiation of reticulocytes into mature red blood cells and named the extruded structures “exosomes”, marking a milestone in the field [4,5]. In 1966, Sun et al. observed dense layered membranes under an electron microscope in large alveolar cells of rats, which were found to be in direct contact with the cell membrane and the alveolar space [6]. Building on these findings, in 1996, Raposo et al. showed that EVs released by B lymphocytes could activate T cells, revealing their role in immune modulation [7].

These pioneering studies have guided subsequent functional investigations. Today, EVs—isolated from various tissues or artificially produced—are utilized across diverse biological fields. These include their roles as drug delivery vehicles, immune modulators, and vectors in regenerative medicine and bioengineering [8,9,10,11,12,13].

As the body’s largest organ, the skin is crucial in protecting the body by acting as a barrier between the internal systems and external environments. It is also essential for sensory perception, thermoregulation, and metabolic functions. Structurally, the skin consists of the epidermis, dermis, and subcutaneous tissue. Skin diseases like psoriasis and atopic dermatitis often cause symptoms like itching and pain. Among these, malignant melanoma significantly affects patient survival rates. However, compared to internal organs, the skin is readily accessible from the outside, offering the opportunity to modulate immune responses through topical treatments. As a result, over the past few decades, extensive research has been conducted on the role of EVs in skin pathogenesis and therapy. This review explored the involvement of EVs in various skin disorders and their potential therapeutic applications, aiming to pave the way for novel treatment strategies in dermatology.

## 2. Background of Extracellular Vesicles: Biogenesis, Classification, and Functions

### 2.1. Biogenesis of Extracellular Vesicles and Classification

#### 2.1.1. Properties of Exosomes

Exosomes, a subtype of small extracellular vesicles, are the smallest type, typically measuring between 40 and 160 nm in diameter [14]. Originating from the endosomal compartment, they are produced through the inward budding of the plasma membrane, ultimately forming multivesicular bodies (MVBs). These MVBs then fuse with the plasma membrane, releasing intraluminal vesicles into the extracellular space. Exosomes transport a wide range of substances, including proteins, nucleic acids (such as DNA, RNA, and microRNAs), lipids, and metabolites, which mediate intercellular communication. In 1987, R.M. Johnstone first introduced the term “exosomes” [4] after observing the release of vesicles during reticulocyte maturation. With their small size and biocompatibility, exosomes are considered ideal carriers for delivering specific molecular cargo to distant cells, making them promising tools for therapeutic interventions and diagnostics.

#### 2.1.2. Properties of Microvesicles

Larger than exosomes, microvesicles (MVs), also referred to as ectosomes, range from 100 to 1000 nm in size. While exosomes originate from the inward budding of late-stage endosomes, MVs are released by the outward budding of the plasma membrane [15]. MVs often transport various cargo, such as proteins, lipids, and RNA, reflecting the surface properties of the parent cell. In the skin, MVs play roles in inflammation, immune responses, and regeneration.

For example, thermal burn injury significantly increases the production of microvesicle particles in HaCaT cells and primary human keratinocytes. The production of MVs is platelet-activating factor receptor (PAFR)-dependent. Platelet-activating factor (PAF) on the cell membrane can be transported via MVs and released into the extracellular environment, thereby regulating the inflammatory response in the skin and other tissues [16].

Another example of MV-mediated communication is the dose-dependent induction of MVs by gemcitabine. This process is regulated by the PAFR and the acid sphingomyelinase (aSMase) pathway. These MVs contain a PAFR agonist, which may influence the therapeutic efficacy of gemcitabine through both local and systemic mechanisms. By targeting the PAFR/aSMase pathway, the effectiveness of gemcitabine in treating skin cancer could potentially be enhanced [17].

#### 2.1.3. Properties of Apoptotic Bodies

Apoptotic bodies (ABs), the largest subtype of EVs, typically have a diameter between 500 and 2000 nm. They form during programmed cell death (apoptosis) when the plasma membrane bulges outward, creating vesicle-like structures that are eventually released into the extracellular space. Their large size and distinct cargo composition, including cellular debris and signaling molecules, make them particularly relevant in cell clearance by phagocytes and immune modulation.

By isolating and reconstructing apoptotic bodies, ABs may specifically target macrophages and cancer cells [18], promote bone healing [19], and facilitate the repair of injured endothelium [20].

For example, *Staphylococcus aureus* (*S. aureus*), a facultative anaerobe with high pathogenicity, could form infection reservoirs in macrophages and cancer cells, thus protecting itself from eradication by systemic antibiotics. However, vancomycin-loaded reconstructed apoptotic bodies (ReApoBds) from cancer cells could effectively eliminate *S. aureus* infections in macrophages and cancer cells [18]. Another study showed that osteoclast-derived ABs share a similar RNA transcriptome with their parent cells. Preosteoclast-derived ABs promoted angiogenesis, while mature osteoclast-derived ABs enhanced bone formation, suggesting their role in coordinating bone remodeling [19]. ABs from mature endothelial cells (ECs) significantly increased the number of human cardiovascular endothelial progenitor cells (EPCs) in vitro. The effect of apoptotic body-rich medium (ABRM) on EPC proliferation is dose-dependent. Additionally, these EC-derived ABs can alter the differentiation status of EPCs, promoting their differentiation into endothelial-like cells [20].

In the skin, ABs are essential for maintaining skin homeostasis. For instance, ABs have been shown to undergo metabolism in the skin and hair follicles. The Wnt/β-catenin pathway is essential for many biological processes in the skin, including stem cell proliferation and differentiation, hair follicle cycling, and wound healing. Through activation of the Wnt/β-catenin signaling pathway, ABs facilitate the progression of these processes [21].

ABs also exhibit a therapeutic role in skin diseases. Stem cell-derived ABs have demonstrated the ability to enhance wound healing and regenerate tissues, particularly those originating from embryonic stem cells. The key mechanisms involve the SOX2/Hippo pathway. Additionally, caspase-3 activation is essential for the production of ABs [22]. Similarly, ABs from mesenchymal stem cells (MSCs) induced macrophage polarization toward the anti-inflammatory M2 phenotype, promoting skin wound healing, formation of granulation tissue, and angiogenesis in mice [23].

### 2.2. Isolation and Identification of Extracellular Vesicles

#### 2.2.1. Isolation of Extracellular Vesicles

Natural EVs can be isolated from various sources, including biofluids (e.g., plasma, urine, saliva, and sweat) and tissues (e.g., skin). The main methods for isolating EVs include differential ultracentrifugation, density gradient centrifugation, size-exclusion chromatography, precipitation kits, and immunocapture [24,25,26,27,28]. To address the issue of data inconsistency across different laboratories, the International Society for Extracellular Vesicles (ISEV) has established the “Minimal Information for Studies of Extracellular Vesicles” (MISEV) guidelines [24]. These guidelines standardize EV isolation, characterization, and functional detection methods.

Differential ultracentrifugation (DUC) is the most widely used method and is considered the gold standard for isolating EVs [29,30]. This technique separates particles primarily based on their size and mass by applying a high centrifugal force. The process involves removing larger vesicles, debris, and cells through a series of centrifugation stages, followed by pelleting the small EVs. DUC yields more EVs compared to size-exclusion chromatography. However, co-isolation of protein aggregates may reduce purity [31]. A washing step after DUC can help remove non-EV components, such as lipoproteins and other plasma proteins [32].

Density gradient ultracentrifugation (DGUC) is another effective method for separating and purifying EVs [33]. By separating samples over a density gradient medium, EVs with similar sizes can be separated based on density. This approach is suitable for applications that require higher purity of EVs [34].

Size-exclusion chromatography (SEC) separates vesicles according to their hydrodynamic radius [35]. This is an easily accessible and rapid approach that helps preserve EV integrity and function [36]. SEC-based methods effectively minimize contamination from proteins that persist when using ultracentrifugation [35]. However, completely separating EV samples by category is challenging due to size overlap.

Precipitation kits offer a simple and quick method. After adding polymers (e.g., polyethylene glycol) to samples, vesicles clump together and precipitate out of solution. EVs can then be harvested by low-speed centrifugation. This method is straightforward but may co-precipitate non-vesicular contaminants [37].

Immunoaffinity capture: EVs have specific surface markers, such as CD9, CD63, and CD81 (tetraspanins). By utilizing antibodies against EV-specific surfaces, this method allows for high specificity and purity [38]. However, it is more expensive and less scalable than other methods.

In addition to classical isolation methods, some optimized alternative approaches or combinations of different techniques are also being developed. For example, tangential flow filtration (TFF) shows higher small EV (sEV) yield, efficiency, and scalability than DUC. TFF combined with SEC is proposed as a more effective and cost-efficient method for isolating EVs [39]. In addition, the combination of UC and a polymer-based precipitation strategy may serve as an effective technique for obtaining small EVs from human serum on a larger research scale [40].

The selection of the isolation method depends on the “donor” cells and on the specific physical properties of the EVs in question. However, as EVs comprise a highly heterogeneous group of vesicles and originate from various sources using diverse isolation methods, clear guidelines and standardized procedures are essential to ensure the reliability and comparability of results across laboratories.

These technical standards have been continuously published under “Minimal Information for Studies of Extracellular Vesicles” in 2014 [41], 2018 [42], and 2024 [24]. In accordance with ongoing scientific progress, the guidelines will be updated further. In the following, the most important parameters for EV characterization are given as a summary.

The protein content of the EVs can be classified into several categories. Transmembrane or glycosylphosphatidylinositol (GPI)-anchored proteins derived from plasma membranes, or endosomes (in eukaryotes), or outer membranes (in prokaryotes), as well as cytosolic or periplasmic proteins that support the presence of enclosed intracellular content, are typically found in EV preparations [43,44]. These proteins are considered “bona fide” markers for EVs. On the other hand, the presence of non-vesicular or secreted proteins (e.g., apolipoproteins, albumin) may indicate sample contamination [42].

Beyond the mere protein detection within EV preparations, the localization of proteins, i.e., membrane-exposed vs. luminal, can be critical for the function of EVs. For instance, proteins encapsulated within the EV lumen require fusion with recipient cells or active transport to exert effects, whereas proteins on the EV surface can act directly on target cells. Biochemical characterization of EVs using techniques such as mild digestion, membrane permeabilization, or antibody-based assays, will determine the localization of functional components [45].

As a third parameter, the cell-contact independence of the EV-mediated functions should be assessed. To demonstrate this, co-culture systems such as transwells or advanced microfluidic devices can be employed to separate EV-donor and EV-recipient cells [46,47].

#### 2.2.2. Identification of Extracellular Vesicles

The main methods for identifying EVs include analyzing their morphology, surface marker expression, and cargo composition.

High-resolution imaging techniques are crucial for observing the size and morphology of EVs at the nanometer scale. Transmission Electron Microscopy (TEM) is considered the gold standard for visualizing EVs [48]. This approach is useful for confirming the presence of EVs and distinguishing them from non-EV particles [49]. For instance, a study by Jin et al. tracked EVs secreted by bone marrow mesenchymal stem cells. TEM imaging revealed that most of these EVs were spherical or elliptical, displayed size variations, and possessed intact capsules, with diameters ranging from 90 to 230 nm. Notably, these EVs were found to have therapeutic potential in treating systemic sclerosis [50]. Additionally, TEM confirms that EVs have been successfully labeled with magnetic nanoparticles, improving their imaging capabilities [51].

Nanoparticle tracking analysis (NTA) is another prominent technique for identifying EVs. Based on the analysis of Brownian motion, NTA is widely used to determine the concentration and size distribution of EVs, particularly in the range from 10 to 1000 nm. The light scattering mode provides precise information about the size, distribution, and concentration of EVs. Meanwhile, the fluorescence mode enables labeling specific protein markers, offering further characterization of EVs [52].

Analyzing the surface markers expressed by EVs is crucial for their phenotypic characterization. The most commonly used exosome markers are tetraspanins, including CD9, CD63, and CD81. Tetraspanins are a superfamily of molecules with four transmembrane domains. They participate in the assembly and recruitment of proteins, lipids, and nucleic acids into EVs [53]. These protein markers can be analyzed by flow cytometry and Western blotting. However, not all EVs express these tetraspanins simultaneously [24]. For a more detailed profiling of EV proteins and cargo, mass spectrometry is the most powerful tool. To complicate matters, EVs from different cell sources often express different surface markers, which can help identify the source of EVs—whether they are isolated from specific tissues or body fluids. For example, MSC-derived EVs (MSC-EVs) consistently express CD44, CD73, and CD105, while CD11b, CD45, and CD197 can serve as reliable negative markers. This helps distinguish MSC-EVs from other EV types [54].

Overall, TEM provides detailed information on size and morphology, NTA offers quantitative analysis and size distribution, and surface marker profiling enables detailed phenotypic characterization. Together, these techniques facilitate a comprehensive understanding and identification of EVs.

#### 2.2.3. Mechanisms of Extracellular Vesicle Uptake

Exosomes enter recipient cells through various pathways. One major endocytic pathway is clathrin-mediated endocytosis, a well-regulated process involving the stepwise assembly of receptors and ligands to form clathrin-coated vesicles. In addition to clathrin-mediated entry, lipid raft- and caveolin-mediated endocytosis also play key roles, with lipid rafts guiding exosomes into early endosomes. Additionally, phagocytosis and macropinocytosis allow for the fluid-phase uptake of larger vesicles. In some cases, exosomes directly fuse with the target cell membrane and release their cargo into the cytosol of target cells [55].

Microvesicles, like exosomes, can be taken up by recipient cells through clathrin/caveolin-mediated endocytosis and macropinocytosis. However, their larger size may favor macropinocytosis. Studies have shown that within the alveolar space, epithelial cells rely exclusively on the clathrin- and caveolin-dependent endocytic pathway, while alveolar macrophages may utilize phagocytosis and scavenger receptors for MV uptake. Phosphatidylserine on the surface of MVs is crucial for their recognition and uptake. The internalization of MVs by epithelial cells can be inhibited by phosphatidylserine-binding proteins such as annexin V [56].

Due to their large size, apoptotic bodies are primarily taken up by phagocytes through the efferocytosis process, which is essential for maintaining tissue homeostasis [57].

#### 2.2.4. Artificially Synthesized Extracellular Vesicles in Comparison to Natural Extracellular Vesicles

Although EVs can play a role in treating various diseases, their isolation, purification, and characterization are complex and prone to errors, as EVs always carry markers of the host cells, which may cause adverse effects. Therefore, scientists have set out to synthesize artificial EVs to customize their functions according to specific needs [58,59,60].

There are two primary ways to construct artificial EVs: the top-down and bottom-up approaches [61].

The top-down approach involves breaking down larger, more complex structures into smaller vesicles to generate nanosized materials. This methodology applies engineering techniques to modify natural cells [62] and create exosome-mimetic models [61]. One example is the application of extracellular vesicle-mimetic nanovesicles (EMNVs) with high yield as a delivery platform for LncRNA-H19. These EMNVs containing LncRNA-H19 (^H19^EMNVs) had similar morphology and surface markers to exosomes and can be internalized by human dermal microvascular endothelial cells (HMEC-1). Under high glucose conditions, ^H19^EMNVs restored the expression of LncRNA-H19 and promoted cell proliferation, migration, and angiogenesis. In vivo, wounds treated with ^H19^EMNVs closed significantly faster than those in the control group, indicating that EMNVs had the potential to treat wounds in diabetic disease [63].

Bottom-up approaches produce EVs from scratch. This process requires lipid-based vesicles (liposomes) or polymer nanoparticles as basic components [61]. Compared with top-down production, bottom-up approaches provide greater control over vesicle design and functionality. Staufer et al. demonstrated the feasibility of bottom-up synthetic methods to construct fully functional synthetic EVs (fsEVs). Synthetic double-stranded miRNA mimics and recombinant tetraspanins CD9, CD63, and CD81 were incorporated into these fsEVs to recreate fibroblast-derived EVs with therapeutic potential. These fsEVs promoted cell proliferation, migration, and collagen deposition, indicating their potential in wound healing [64].

Unlike natural EVs, which face challenges in large-scale production, artificially synthesized EVs enable both high-yield generation and scalability [65]. They offer great flexibility regarding the cargo they transport and the surface molecules they express. For example, surface markers can be customized to target different cells efficiently. However, artificially synthesized EVs may induce immunogenicity due to the artificial components in the manufacturing process, such as the composition of lipid layers and the density and variability of surface molecules.

Therefore, engineering strategies combining natural and artificially synthesized EVs may harness both advantages, potentially enhancing therapeutic efficacy.

#### 2.2.5. Modified Extracellular Vesicles and Applications in Drug Delivery

Engineering EVs makes it possible to target specific cells or tissues, thereby enhancing their therapeutic efficacy and enabling more precise drug delivery. Modification of EVs can be achieved by altering their parent cells, and direct modification of the EVs is also feasible.

Targeted therapy has been widely explored in the field of oncology. As an example, advanced melanoma is a severe and rapidly progressing skin cancer with a low five-year survival rate, emphasizing the importance of developing novel therapeutic solutions. The combination of EVs and adenovirus-based vaccines can achieve targeted therapeutic effects. Mathlouthi et al. combined oncolytic adenovirus with a specific melanoma antigen (NRAS mutation-specific antigen) and encapsulated this adenovirus-based cancer vaccine within EVs. They evaluated its therapeutic efficacy against melanoma. The study showed that the EV-encapsulated adenovirus vaccine reduced tumor volume and increased immune cell infiltration. Furthermore, the EV-encapsulated adenovirus vaccine accumulation in tumor tissues was significantly higher than that of free adenovirus. This demonstrates that the method improves the local concentration and duration of the vaccine, allowing for targeted tumor therapy. Additionally, EVs protect their cargo (such as adenovirus) from recognition and clearance by the host immune system. This extends the half-life of the vaccine in the body and enhances its therapeutic efficacy [66]. The lung is one of the primary target organs for metastasis of B16-BL6 cells, a murine melanoma cell line. In one study, EVs were isolated from B16-BL6 melanoma cells and used to coat the surface of the organosilica nanocages (ssOSCs). Subcutaneous tumor and metastasis models were established in C57BL/6 mice. Doxorubicin (DOX) was loaded into the ssOSCs, followed by their subsequent coating with EV membranes (ssOSCs-EVs^B16-BL6^). The results showed that ssOSCs-EVs^B16-BL6^ predominantly accumulated in the lungs, with much lower accumulation in the liver and spleen. This suggests that ssOSCs-EVs^B16-BL6^ can enhance drug concentration at tumor sites, reduce potential toxicity in the liver and spleen, and thereby minimize systemic side effects [67].

However, in the context of inflammatory skin diseases, there is a lack of extensive research on surface modification of EVs to achieve targeted delivery to the affected inflammatory sites. The primary strategy involves targeting specific signaling pathways. For example, one study loaded the Arginase-1 (Arg1) inhibitor nor-NOHA into MSC-EVs to create engineered nor@MSC-EVs and then evaluated their therapeutic effects in psoriasis. Arg1 is overexpressed in the epidermis of psoriasis patients and correlates with disease severity. The engineered nor@MSC-EVs inhibited the NF-κB pathway by targeting the Arg1/polyamine-mediated dendritic cells (DCs)/ T helper 17 (Th17) axis, reducing T helper 1 (Th1) and Th17 cell differentiation and alleviating skin lesions in a psoriasis mouse model [68]. In another study, miR-21-5p mimics were loaded into human adipose-derived, stem cell-derived exosomes using electroporation. These engineered EVs enhanced miR-21-5p uptake. They promoted HaCaT cell proliferation and migration in vitro and accelerated wound healing in diabetic rats in vivo by activating the Wnt/β-catenin signaling pathway [69].

In other inflammatory diseases, such as rheumatoid arthritis, surface modification of EVs enables specific targeting of molecular receptors. For example, by introducing azide groups onto the surface of cells and conjugating them with dibenzocyclooctyne-terminated PEGylated hyaluronic acid (DBCO-PHA), PHA-decorated EVs (PHA-EVs) were successfully constructed. These PHA-EVs had CD44 receptor-targeting capabilities. In the collagen-induced arthritis mouse model, the fluorescence signal of the PHA-EVs at inflamed joints in rheumatoid arthritis was significantly stronger and more sustained compared to that of unmodified EVs, indicating their potential as a novel nanotherapeutic for the treatment of inflammatory diseases [70]. This provides a potential approach for investigating skin diseases. Surface modification of EVs could allow them to target specific receptors on skin cells. For example, the DEC-205 receptor is found on dendritic cells, directing antigens into deeper endocytic vesicles containing MHC class II molecules [71]. Targeting DEC-205 may enable EVs to effectively target DCs and facilitate the presentation of their contents by DCs.

## 3. Extracellular Vesicles and Psoriasis

Psoriasis is a chronic immune-mediated inflammatory skin disease characterized by erythema, scaling, and abnormal proliferation of keratinocytes. The worldwide prevalence is about 2% [72]. Psoriasis affects not only the skin but also the joints and various organ systems [73]. Although psoriasis generally does not impact survival, its symptoms can cause aesthetic and psychological distress, significantly reducing life quality of patients.

### 3.1. Pathology of Extracellular Vesicles in Psoriasis

The pathogenesis of psoriasis is complex and not yet fully understood. The immune system plays a central role, with key players including T cells (particularly Th1 and Th17 cells), DCs, and cytokines such as IL-23, IL-17, TNF-α, and IFN-γ. Th17 cells are especially crucial, as they produce IL-17 and IL-22, which drive keratinocyte proliferation and inflammation.

EVs are secreted by various cell types, including keratinocytes [74], fibroblasts [75,76], skin-related stem cells [77], macrophages [78], and dendritic cells [79,80]. They facilitate communication between keratinocytes and immune cells, transmit psoriasis-related inflammatory signals, and promote inflammation and disease progression [81].

#### 3.1.1. Extracellular Vesicles Induce Release of Proinflammatory Molecules

**Figure 1:**
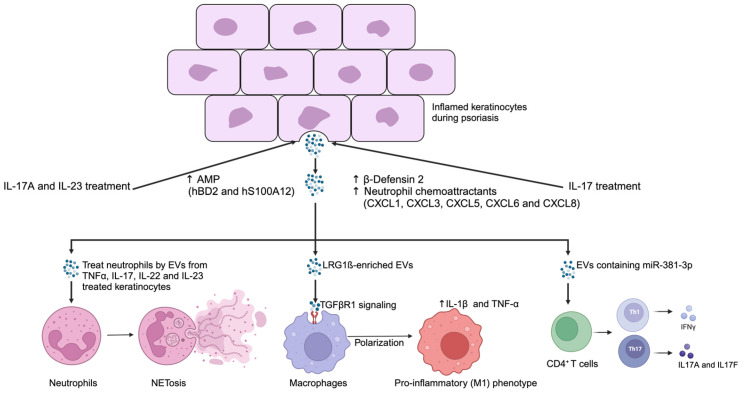
Keratinocyte-derived EVs modulate immune cells and promote inflammation in psoriasis. EVs released from keratinocytes treated with recombinant IL-17A (rIL-17A) and IL-23 (rIL-23) exhibit elevated levels of antimicrobial peptide (AMP) mRNAs, such as human β-defensin 2 (hBD2) and S100A12 [83]. Long-term IL-17A exposure further enhances β-defensin 2 expression and increases mRNA levels of neutrophil chemoattractants within EVs [82]. EVs from psoriasis-related, cytokine-treated keratinocytes can induce neutrophil extracellular trap formation (NETosis) in neutrophils [83]. Keratinocyte-derived EVs enriched with leucine-rich α-2-glycoprotein 1 (LRG1) promote M1 macrophage polarization via the TGFβR1 signaling pathway, leading to upregulation of pro-inflammatory genes [81]. EVs from inflamed keratinocytes transfer miR-381-3p to CD4^+^ T cells, promoting Th1 and Th17 polarization and thereby contributing to psoriasis progression [74]. ↑: Upregulation, activation, or increased expression, activity, or level.

Previous studies conducted by Mangino et al. have shown that treatment with IL-17A, a key cytokine in psoriasis, reduces EV release by keratinocytes (HaCaT cells). However, these EVs induce recipient cells to express psoriasis-related mRNAs, such as β-Defensin 2, suggesting their involvement in disease progression [82] (Figure 1).

Capriotti et al. further validated that psoriasis-related cytokines like IL-17A, IFN-γ, and IL-22 influence the release and content of EVs from keratinocytes. These EVs transport inflammatory molecules, including antimicrobial peptides (AMPs) such as hBD2 and S100A12, contributing to psoriasis-related inflammation. Additionally, EVs derived from HaCaT cells can induce neutrophil extracellular trap formation (NETosis) in neutrophils, triggering the release of pro-inflammatory cytokines such as IL-17A and TNF-α. Both cytokines play a central role in psoriasis pathogenesis [83].

Interestingly, Mangino et al. [82] mentioned that treating human primary keratinocytes with rIL-17A reduced EV release, whereas Capriotti et al. [83] reported that IL-17A and IFN-γ enhanced EV secretion from keratinocytes. This discrepancy may be attributed to the different cell models used in these studies. Mangino et al. [82] used human primary keratinocytes, while Capriotti et al. [83] investigated using HaCaT cell lines. Notably, HaCaT cells are immortalized keratinocytes that differ from primary keratinocytes and may not fully replicate the natural physiological state of the skin in vivo.

#### 3.1.2. Extracellular Vesicles Promote Th1/Th17 Polarization

Polarization of T helper cells (Th1 and Th17) drives inflammation in psoriasis. Compared with sEVs derived from untreated keratinocytes, EVs from anti-CD3 and anti-CD28-stimulated keratinocytes significantly upregulate the mRNA levels of Th1- and Th17-related cytokines in T cells. Additionally, miR-381-3p is highly expressed in treated sEVs. miR-381-3p mimics can significantly upregulate the expression of Th1- and Th17-related cytokines and respective transcription factors, while inhibitors can suppress these effects. Thus, these data indicate that EVs derived from keratinocytes promote Th1/Th17 polarization by transferring miR-381-3p, promoting psoriasis progression [74] (Figure 1).

#### 3.1.3. Extracellular Vesicles Trigger Macrophage Polarization

Jiang et al. treated HaCaT cells with M5, a combination of cytokines including IL-17A, IL-22, TNF-α, IL-1α, and Oncostatin M. The supernatant from these HaCaT cells significantly increased the expression of IL-1β, TNF-α, CD86, and IL-10 in THP-1 cells, a monocytic cell line. This finding suggests that EVs derived from stimulated HaCaT cells are pivotal in promoting macrophage polarization toward the M1 phenotype [81]. M1 macrophages are known to upregulate matrix metalloproteinases (MMPs) [84], which are crucial for extracellular matrix remodeling. However, excessive MMP activity contributes to skin barrier disruption and tissue damage [85,86]. To further validate the role of EVs, the researchers treated keratinocytes with an inhibitor that blocks EV secretion. This intervention significantly reduced IL-1β and TNF-α levels in co-cultured THP-1 cells, reinforcing the idea that EVs are key mediators of macrophage activation by keratinocytes. Moreover, these EVs were found to be enriched with leucine-rich α-2-glycoprotein 1 (LRG1), which activates macrophages through the TGF beta receptor 1 (TGFβR1)-dependent pathway [81] (Figure 1).

#### 3.1.4. Extracellular Vesicles and Psoriasis-Associated Conditions

EVs also play a crucial role in psoriasis-associated conditions, including psoriatic arthritis (PsA) [87]. PsA affects the musculoskeletal system and is frequently associated with comorbidities such as cardiovascular disease, metabolic syndrome, obesity, diabetes, and inflammatory bowel disease. In a study by Lättekivi et al., the miRNA profiles and surface proteomes of EVs in the serum of patients with psoriasis vulgaris (PsV) and PsA were analyzed and compared with those of healthy controls. The researchers identified several differentially enriched EV-bound miRNAs linked to disease pathology. Moreover, EV array analysis revealed a significant enrichment of the EV marker CD9 in PsV patient samples [88].

### 3.2. Therapeutic Applications of Extracellular Vesicles in Psoriasis

#### 3.2.1. Extracellular Vesicles as Diagnostic and Prognostic Biomarkers for Psoriasis

EVs can be isolated from various biofluids, including blood, sweat, and urine, offering a non-invasive approach for diagnosing and monitoring skin diseases.

In psoriasis, circulating exosomes isolated from patient plasma have been found to contain elevated levels of pro-inflammatory mediators such as IL-17A [89], miR-146a [90], miR-31-5p, miR-7-5p, miR-146a-3p, miR-944, miR-21-3p, miR-147b, miR-431-5p, miR-3614-5p, miR-223-5p [91], miR-625-3p, miR-4488, and miR-342-3p [92], compared to healthy controls.

Psoriasis severity is often assessed using clinical indices such as the Body Surface Area (BSA) and the Psoriasis Area and Severity Index (PASI), classifying the disease as mild, moderate, or severe. EVs can serve as potential biomarkers not only for disease presence but also for severity stratification. For example, Park et al. demonstrated that miR-625-3p levels were significantly associated with both PASI and BSA scores, highlighting its diagnostic value in distinguishing mild-to-moderate from moderate-to-severe psoriasis [92]. Similarly, miR-147b and miR-3614-5p were significantly elevated in patients with high PASI scores compared to those with lower scores [91].

Interestingly, while some EV-associated miRNAs correlate positively with psoriasis severity, others show negative correlations. For instance, reduced levels of let-7b-3p, miR-1181, miR-125b-5p, miR-1268a, miR-499b-3p, and miR-877-3p have been observed in more severe cases [93]. Furthermore, plasma EV levels of let-7b-5p and miR-30e-5p were significantly lower in patients with PsA compared to those with cutaneous-only psoriasis [87].

Collectively, these findings suggest that EVs, particularly their miRNA cargo, hold promise as sensitive and specific biomarkers for the diagnosis, severity assessment, and prognosis of psoriasis.

#### 3.2.2. Immune Regulation and Anti-Inflammatory Actions

In inflammatory skin conditions such as psoriasis, immune system dysregulation leads to chronic inflammation, erythema, and epidermal thickening. EVs derived from various cell types have emerged as promising tools to modulate immune responses and exert anti-inflammatory effects.

##### Naturally Derived Extracellular Vesicles

MSCs derived from sources such as umbilical cord blood (UCB) and adipose tissue are widely used in regenerative medicine. Importantly, EVs derived from MSCs have been shown to recapitulate many therapeutic effects attributed to the parental MSCs, owing to their rich cargo of lipids, proteins, and nucleic acids.

In a clinical study, exosomes isolated from healthy adipose-derived MSCs significantly reduced erythema, induration, and skin thickness in psoriasis patients. A 200 µg dose led to a notable reduction in inflammatory cytokines such as IL-23, TNF-α, IL-17, and IFN-γ. Concurrently, anti-inflammatory mediators such as IL-10 and FOXP3 were upregulated, supporting the potential of MSC-derived exosomes as a promising cell-free therapeutic strategy for psoriasis [94]. Additionally, Zhang et al. demonstrated that IFN-γ-stimulated MSC-derived EVs (IFNγ-sEVs) inhibited T cell proliferation, suppressed inflammatory cytokine production, and alleviated psoriasis-like symptoms in a murine model [95]. Another study found that UCB-derived mononuclear cell EVs (UCB-MNC-sEVs) induced macrophage polarization toward an anti-inflammatory phenotype, reduced CD4^+^ and CD8^+^ T cell proliferation, and downregulated psoriatic markers, including IL-6, IL-8, CXCL10, COX2, S100A7, and DEFB4 [96].

Collectively, these findings suggest that MSC-derived EVs have significant therapeutic potential for treating psoriasis and other inflammatory skin diseases.

##### Engineered Extracellular Vesicles

EVs can also be bioengineered to enhance their therapeutic functions.

Bacterial extracellular vesicles (bEVs) have emerged as critical mediators of host–microbiota interactions. A recent study reported that oral administration of outer membrane vesicles (OMVs) derived from *Parabacteroides goldsteinii* (Pg OMVs), which can be mass-produced, alleviated epidermal hyperplasia and reduced skin inflammation in a murine model of psoriasis. Pg OMVs were shown to traverse the intestinal barrier and reach inflamed skin, suppressing immune cell infiltration and systemic inflammation [97].

Another innovative approach utilized *Cutibacterium acnes*, a commensal skin bacterium. Its EVs were encapsulated within GelMA hydrogel microspheres (CA-EVs@GHM) to allow for sustained release. In vitro, CA-EVs@GHM promoted HaCaT cell proliferation and migration, enhanced resistance to *Staphylococcus aureus*, and downregulated IL-6 and CXCL8 levels. In an imiquimod (IMQ)-induced psoriasis mouse model, treatment with CA-EVs@GHM suppressed pro-inflammatory cytokines (TNF, IL-6, IL-17A, IL-22, IL-23A), restored skin barrier function and microbial diversity, reduced *S. aureus* colonization, and inhibited ILC2-to-ILC3 conversion—ultimately reducing IL-17 and IL-22 secretion [98].

Interestingly, tumor-derived exosomes have also been engineered for psoriasis therapy. During psoriatic inflammation, both keratinocytes and immune cells express PD-1. Jia et al. isolated PD-L1^+^ exosomes from melanoma cells and loaded them with Pristimerin, a natural anti-inflammatory compound. These engineered exosomes targeted PD-1^+^ keratinocytes and immune cells, inhibiting Th17 cell proliferation, promoting regulatory T cell (Treg) differentiation, reducing immune cell infiltration, suppressing pro-inflammatory cytokine production, and alleviating psoriatic symptoms in a mouse model [99].

The derivation of EVs and their therapeutic effects of EVs in psoriasis are summarized in Table 1.

## 4. Extracellular Vesicles and Atopic Dermatitis

### 4.1. Pathology of Extracellular Vesicles in Atopic Dermatitis

Atopic dermatitis (AD), commonly known as eczema, is a chronic inflammatory skin disorder characterized by symptoms such as pruritus, xerosis, and cutaneous inflammation [100]. The pathogenesis of AD is multifactorial, involving a complex interplay between genetic predisposition, microbial influences, skin barrier dysfunction, and immune dysregulation [101,102]. A hallmark of AD is the imbalance between type 1 and type 2 helper T cells (Th1/Th2), with a predominance of Th2-mediated immune responses. Th2 cytokines stimulate B cells to produce IgE, which activates mast cells and triggers degranulation. Additionally, cytokines such as IL-22 and IL-17, secreted by Th22 and Th17 cells, respectively, contribute to skin barrier damage and disease progression [103].

#### 4.1.1. Genetic Abnormality

The *filaggrin (FLG)* gene is one of the most important susceptibility genes for AD [104]. *FLG* encodes the filaggrin protein, which plays a critical role in skin barrier integrity by promoting keratin filament aggregation and maintaining the skin’s resistance to transepidermal water loss, allergens, and pathogens [105].

In the context of *FLG* deficiency, keratinocyte-derived EVs exhibit marked alterations in lipid composition. Specifically, these EVs contain increased levels of long-chain saturated fatty acids and reduced levels of long-chain polyunsaturated fatty acids. This shift in lipid content impairs the generation of self-antigens suitable for CD1a presentation on antigen-presenting cells, thereby Th1 immune responses and skewing the immune system toward a Th2 bias. Notably, EVs from *FLG*-deficient keratinocytes were found to suppress CD1a-mediated IFN-γ production while enhancing IL-13 secretion, further contributing to the Th2-dominant inflammation characteristic of AD [106].

#### 4.1.2. Microbial Influence

Microbial allergens play a pivotal role in the pathogenesis and exacerbation of AD. Common microbial triggers include *Staphylococcus aureus* and fungal species such as *Malassezia*. These organisms penetrate the compromised skin barrier in AD and provoke immune responses, particularly favoring Th2 polarization.

*Staphylococcus aureus* is a key exacerbating factor in AD, with frequent colonization observed in lesional skin [107]. For the first time, Hong et al. identified *Staphylococcus aureus*-derived extracellular vesicles (SA-EVs) on the skin of AD patients. These vesicles significantly contribute to AD-associated inflammation by stimulating dermal fibroblasts to produce pro-inflammatory mediators such as eotaxin, thymic stromal lymphopoietin (TSLP), IL-6, and macrophage inflammatory protein-1α (MIP-1α). These factors promote the activation of both Th2 and Th17 immune pathways, exacerbating inflammation. Cutaneous application of SA-EVs has been shown to induce IgE production [108].

SA-EVs deliver virulence factors, such as staphylococcal protein A (SPA), to keratinocytes, stimulating the release of pro-inflammatory cytokines including IL-6, IL-8, MIP-1α, and MCP-1 [107]. Furthermore, treating human dermal microvascular endothelial cells (HDMECs) with SA-EVs increases IL-6 expression, possibly supporting Th17 differentiation. SA-EVs also enhance the expression of adhesion molecules such as E-selectin, ICAM-1, and VCAM-1 on HDMECs, likely via activation of the Toll-like receptor (TLR) 4 and NF-κB signaling pathways. This upregulation facilitates monocyte adhesion and infiltration, amplifying the local inflammatory response [109].

In primary human keratinocytes (PHKs), SA-EVs induce the expression of CXCL8 in a TLR2- and NF-κB-dependent manner, promoting neutrophil recruitment and NETosis. This in turn supports further *S. aureus* colonization [110].

SA-EVs carry over 100 virulence-associated proteins, including α-hemolysin, SPA, and β-lactamases. These factors disrupt the skin barrier through various mechanisms. For instance, α-hemolysin embedded in EVs forms pores in keratinocyte membranes, inducing necrosis—a process more cytotoxic than apoptosis induced by its soluble form. This destruction of keratinocytes compromises epidermal integrity and allows deeper penetration of pathogens [111]. Additionally, SA-EVs can contribute to antibiotic resistance. They carry β-lactamase (BlaZ), an enzyme that hydrolyzes β-lactam antibiotics such as penicillins and cephalosporins. The dissemination of BlaZ via SA-EVs promotes antibiotic resistance and may contribute to the persistence of chronic infections associated with AD [112].

Another relevant microbial allergen in AD is *Malassezia*, a common commensal fungus on human skin. In AD patients, however, it can act as a pro-inflammatory agent. *Malassezia*-derived extracellular vesicles (MalaEx) contain small RNAs and allergens that are internalized by both keratinocytes and monocytes, localizing near the nucleus. This suggests an active role in initiating and maintaining inflammatory responses.

MalaEx contain small RNAs and allergens internalized by keratinocytes and monocytes, localizing near the nucleus. This suggests an active role in initiating and maintaining inflammatory responses [113].

MalaEx also modulate immune function by increasing the expression of ICAM-1 (CD54) in keratinocytes, facilitating immune cell recruitment to the skin [114]. Moreover, MalaEx stimulate peripheral blood mononuclear cells (PBMCs) from AD patients to secrete elevated levels of IL-4 and TNF-α. The IL-4 response was significantly higher compared to healthy controls, underscoring the Th2-skewing effects of MalaEx [115]. These findings highlight the role of fungal EVs in promoting immune dysregulation and skin barrier degradation in AD.

### 4.2. Therapeutic Applications of Extracellular Vesicles in Atopic Dermatitis

Currently, AD is primarily treated with glucocorticoids, calcineurin inhibitors, and antihistamines. However, many of these medications are unsuitable for long-term use due to limited efficacy and potential side effects. EVs offer a promising alternative by enhancing skin barrier function and modulating immune responses to reduce inflammation.

#### 4.2.1. Stem Cell-Derived Extracellular Vesicles for Atopic Dermatitis Treatment

MSCs play a crucial role in tissue regeneration. In one study, canine MSCs were encapsulated within calcium alginate nanogels, which improved the stability and targeted delivery of their EVs. These nano-encapsulated EVs effectively regulated cytokines associated with skin inflammation in AD, including IL-2, IL-4, IL-5, IL-6, IL-10, IL-13, and IL-31 [116]. Genetic modification can further enhance the therapeutic potential of MSCs. For instance, MSCs engineered to express extracellular superoxide dismutase 3 (SOD3), an antioxidant enzyme, significantly reduced inflammation-like symptoms in an AD mouse model. These SOD3-MSCs also modulated immune cell activation and differentiation, further alleviating AD symptoms [117].

#### 4.2.2. Bacteria-Derived Extracellular Vesicles for Atopic Dermatitis Treatment

A previous study comparing the levels of EVs in the urine of AD patients and healthy individuals found that *Lactococcus*, *Leuconostoc*, *Lactobacillus* and *Lactobacillales(o)* were more abundant in the control group. Moreover, in vitro experiments showed that treatment of *Lactobacillus plantarum*-derived EVs (LpEVs) before SA-EVs could reduce IL-6 secretion in keratinocytes and macrophages and restore cell viability. In a mouse model, oral administration of LpEVs alleviated SA-EV-induced skin inflammation, reduced epidermal thickness, and decreased IL-4 levels [118].

*Limosilactobacillus fermentum* SLAM216 (LF216) is a specific strain of lactic acid bacteria known to support intestinal health and maintain microbiota balance. A recent study reported that EVs derived from LF216 (LF216-EVs) promoted wound healing in HaCaT keratinocyte cultures and downregulated several pro-inflammatory cytokines, including thymic stromal lymphopoietin (TSLP), TNF-α, IL-6, IL-1β, and macrophage-derived chemokine (MDC). In vivo, treatment with LF216-EVs in a DNCB-induced AD mouse model significantly alleviated AD symptoms. The anti-inflammatory effects of LF216-EVs may be mediated by modulation of serotonin synthesis via changes in the gut microbiota and metabolome, resulting in reduced scratching behavior and improvements in depression-related symptoms in the AD model [119].

#### 4.2.3. Marine- and Plant-Derived Extracellular Vesicles for Atopic Dermatitis Treatment

Interestingly, EVs derived from certain plants and marine organisms also demonstrate therapeutic potential. *Pinctada martensii* (*P. martensii*), a mollusk known for producing pearls, has been investigated in this context. A recent study using HaCaT keratinocytes as an in vitro model for AD found that EVs isolated from *P. martensii* mucus significantly reduced reactive oxygen species (ROS) levels and lysosomal rupture. These EVs also decreased lactate dehydrogenase (LDH) release, indicating notable anti-inflammatory effects. miRNA-seq analysis revealed that miR-100-5p was highly expressed in the EVs and inhibited FOXO3/NLRP3 signaling, thereby reducing inflammation and pyroptosis. Furthermore, in an AD mouse model, combining these EVs with a self-cross-linking hydrogel composed of oxidized sodium alginate (OSA) and carboxymethyl chitosan (CMCS)-enhanced skin healing. This combination therapy significantly reduced skin inflammation and thickness while restoring collagen more effectively than the hydrogel alone [120].

The derivation of EVs and their therapeutic effects in AD is summarized in Table 2.

## 5. EVs and Systemic Lupus Erythematosus

Systemic lupus erythematosus (SLE) is a systemic autoimmune disease characterized by inflammation and immune-mediated damage to multiple organ systems, including the kidneys, musculoskeletal, hematologic, and mucocutaneous systems [121]. The pathogenesis of SLE remains incompletely understood. The primary pathological mechanisms involve the production of autoantibodies targeting nuclear antigens, the formation of immune complexes, and subsequent tissue damage.

### 5.1. Pathology of Extracellular Vesicles in Systemic Lupus Erythematosus

#### 5.1.1. Extracellular Vesicles as Providers of Autoantigens

Nuclear antigens, such as nucleosomal DNA and histones, are normally confined to the nucleus and remain hidden from the immune system under healthy conditions. However, in SLE, dysregulated apoptosis and impaired clearance of apoptotic cells lead to the translocation of these nuclear antigens to the cytoplasm, where they are incorporated into apoptotic bodies. Once released into the extracellular space, these highly immunogenic antigens act as autoantigens, triggering the production of autoantibodies.

#### 5.1.2. Extracellular Vesicles Trigger Immune Response and Immune Complex Formation

When isolating EVs from SLE patients and healthy controls (HCs), higher levels of pro-inflammatory cytokines such as IFN-α, TNF-α, IL-1β, and IL-6 are observed in SLE patients. This inflammatory response occurs in a TLR-dependent manner, suggesting that EVs may act as messengers, spreading inflammation throughout the body and contributing to the systemic consequences of SLE [122]. EVs from SLE patients also carry higher concentrations of IgG, further enhancing their ability to stimulate the immune system and increase inflammation [123]. Moreover, Karlsson et al. found that monomeric C-reactive protein (mCRP) on EVs is significantly elevated in patients with active SLE, indicating that mCRP acts as both an antigen and an adjuvant, promoting the production of anti-CRP autoantibodies and amplifying inflammation [124]. The production of autoantibodies results in immune complex (IC) formation, which deposits in the kidneys and causes lupus nephritis (LN) [125].

#### 5.1.3. Extracellular Vesicles Contribute to Damage to Other Organs in Systemic Lupus Erythematosus

Lupus nephritis is a major cause of morbidity and mortality in SLE. During this process, EVs carry adhesion and costimulatory molecules, leading to IC deposition on kidney endothelial cells [123]. Early studies have shown that, compared with healthy individuals, SLE patients exhibit significantly different microparticle concentrations and compositions. Two distinct annexin V-nonbinding MP subpopulations (AnxV^−^) are significantly elevated in SLE patients, suggesting potential links to SLE activity and lipid metabolism, contributing to kidney disease and cardiovascular risk factors [126]. Another serious complication of SLE is pulmonary arterial hypertension (PAH). In SLE-PAH patients, there is an increase in EVs derived from leukocytes (LEVs), platelets (PEVs), red blood cells (REVs), endothelial cells (EEVs), and Annexin V^+^ EVs. This indicates that EVs contribute to blood clotting in SLE-PAH patients. Elevated EV levels in PAH are significant for early detection and monitoring of SLE-PAH, assisting in disease management and controlling the progression of SLE [127].

### 5.2. Therapeutic Applications of Extracellular Vesicles in Systemic Lupus Erythematosus

#### 5.2.1. Extracellular Vesicles as Diagnostic and Prognosis Biomarkers for SLE

EVs offer a non-invasive approach for diagnosing SLE, distinguishing patients from healthy individuals, and monitoring treatment efficacy.

A plasmonic nanoparticle-embedded polydopamine substrate, modified with EV-capturing molecules and detection probes, enables efficient EV detection. In untreated SLE patients, blood levels of miRNA-146a and PD-1 are lower, while sialic acid (SA) is elevated. In urine, miRNA-146a is increased, while SA remains unchanged. This suggests that urinary miRNA-146a may reflect disease activity, while variations in PD-1 and SA levels in the blood indicate immune system abnormalities [128]. EVs also play a role in pulmonary arterial hypertension (PAH) associated with SLE, enabling early disease detection and monitoring [127]. Circulating EVs are significantly elevated in SLE patients, with or without nephritis, compared to healthy controls. Additionally, SLE patients exhibit larger and chemically distinct EVs with higher CD45, CD14, and IgM levels, indicating an immune cell origin [129].

#### 5.2.2. Extracellular Vesicles Regulate Immune Response

Studies have shown that exosomes derived from bone marrow mesenchymal stem cells (BMMSCs) can modulate macrophage polarization toward an anti-inflammatory phenotype. In the MRL/lpr mouse model of SLE, exosome treatment reduced CD86 and increased CD206 expression in renal macrophages, along with lowered levels of pro-inflammatory cytokines (IFN-γ, IL-1β, IL-6) and elevated levels of anti-inflammatory cytokines (IL-10, TGF-β). Exosome-treated macrophages also showed enhanced phagocytosis of apoptotic cells and promoted Treg differentiation while reducing the proportion of Th17 cells, indicating broad immunoregulatory effects [130]. Similarly, Dou et al. demonstrated that MSC-derived EVs could alleviate SLE-associated inflammation by suppressing macrophage M1 polarization. This effect was closely associated with tRNA-derived fragments (tRFs), particularly tsRNA-21109. Upregulated in MSC-EV-treated macrophages, tsRNA-21109 targeted multiple inflammation-related pathways (e.g., MAPK, TGF-β, Wnt), downregulated M1 markers, and enhanced M2 polarization [131].

In addition to their effects on macrophages, EVs have also been shown to influence T cells. One study demonstrated that exosomes derived from umbilical cord blood MSCs could effectively regulate the Th17/Treg balance in CD4^+^ T cells from patients with SLE. Specifically, following uptake by T cells, exosomes significantly enhanced the expression of miR-19b, which subsequently downregulated its target gene, KLF13. This led to the inhibition of Th17 cell differentiation and an increase in the proportion of Treg cells. Moreover, there was a marked reduction in the expression of pro-inflammatory cytokines such as TNF-α, IL-6, and IL-17, accompanied by an increase in anti-inflammatory cytokines like IL-10 and TGF-β [132].

Apoptotic extracellular vesicles (apoVs) derived from MSCs also exert immunomodulatory effects in SLE. In MRL/lpr mice, systemic administration of apoVs reduced lymphoproliferation, decreased IFN-γ^+^ CD4^+^ T cells, increased Foxp3^+^ regulatory T cells, and alleviated the inflammatory activity of lupus and arthritis. ApoVs suppressed CD4^+^ T cell activation in a dose-dependent manner, particularly affecting Th1 and Th17 subsets through direct contact independent of macrophages. The mechanisms underlying these effects are primarily mediated through phosphatidylserine on apoVs, which interacts with the TIM-3 receptor on T cells, thereby interfering with T cell receptor signaling [133].

Interestingly, EVs derived from stem cells of human exfoliated deciduous teeth (SHED-EVs) also showed therapeutic effects in treating SLE. Systemic injection of SHED-EVs improved peripheral autoantibody levels, renal function, and immune status in MRL/lpr mice. RNase treatment attenuated the therapeutic effects of SHED-EVs, suggesting that RNA secreted by SHED played a crucial role in this process. Additionally, SHED-EVs could be taken up by recipient BMMSCs, restoring Telomerase Reverse Transcriptase (Tert) expression and telomerase activity, which enhanced the hematopoietic niche formation function of BMMSCs [134].

The derivation of EVs and their therapeutic effects in SLE is summarized in Table 3.

## 6. EVs in Skin Wound

### 6.1. Pathology of Extracellular Vesicles in Skin Wound

Mitochondria-derived vesicles (MDVs) are secreted by mitochondria to facilitate the removal of damaged mitochondrial components. Zhang et al. discovered elevated levels of MDVs in the skin tissue of patients with diabetic foot ulcers (DFUs). Under high glucose conditions, researchers isolated secreted MDVs (hgMDVs) from fibroblasts. Through proteomic analysis, they found that hgMDVs had a similar protein composition to MDVs in DFU tissues. Further experiments demonstrated that hgMDVs significantly promoted apoptosis and oxidative stress, disrupted mitochondrial structure, and reduced aerobic metabolism, ultimately delaying wound healing in diabetes [135].

### 6.2. Therapeutic Applications of Extracellular Vesicles in Skin Wound Healing

#### 6.2.1. Extracellular Vesicles Enhance Cell Proliferation and Migration

EVs from adipose-derived stem cells promote fibroblast proliferation, migration, and collagen production. When combined with hyaluronic acid (HA), these EVs improved wound closure, enhanced re-epithelialization, and increased collagen type III deposition in a porcine wound model, compared to HA alone [136].

Macrophage-derived EVs transfer bioactive molecules, including miR-425-5p, to skin fibroblasts. This promotes adipokine expression, keratinocyte proliferation, and wound repair [137].

Platelet-derived EVs (pEVs) contain platelet membrane proteins and growth factors such as IGF and TGF-β, which aid wound healing. In vitro, pEVs enhanced fibroblast proliferation, migration, and endothelial tube formation, supporting angiogenesis for better oxygen and nutrient delivery. In a Phase I clinical trial, healthy volunteers were randomly assigned to receive either an injection of 100 μg Ligand-based Exosome Affinity Purification (LEAP)-purified pEVs or a placebo. As the first human study to invesigate pEVs as a potential therapy for delayed wound healing, LEAP-purified pEVs showed safety. Wounds in both the treatment and placebo groups healed completely within 30 days, although no significant differences were observed. This may be due to the rapid healing capacity of healthy individuals. Further studies need to be conducted in a chronic wound setting, and the sample size needs to be increased [138].

#### 6.2.2. Extracellular Vesicles Promote Angiogenesis

Wu et al. identified serum- and glucose-deprived EVs (SGD-EVs) from human umbilical cord mesenchymal stem cells (HUCMSCs) that promote skin regeneration and wound healing. SGD-EVs enhanced endothelial cell migration, proliferation, and tube formation, accelerating angiogenesis in a rat wound model. miR-29a-3p, highly enriched in SGD-EVs, activated Wnt/β-catenin signaling to boost VEGFA production and vessel formation [139].

Long et al. demonstrated that MSC-EVs, combined with a 7-amino-acid peptide (7A), significantly improved diabetic wound healing. While 7A alone had no effect, MSC-EVs promoted fibroblast proliferation. Their combination further enhanced healing by inhibiting NF-κB-mediated inflammation, upregulating TGF-β for fibroblast proliferation and collagen synthesis, and increasing CD31 to support angiogenesis [140].

In another study, EVs derived from epidermal stem cells were loaded with VH298 (VH-EVs) and delivered to endothelial cells. VH-EVs were shown to promote the function of human umbilical vein endothelial cells (HUVECs), enhancing wound healing and angiogenesis in diabetic mice. VH-EVs were further incorporated into a gelatin methacryloyl (GelMA) hydrogel, enabling sustained release and showing more epithelialization and collagen fiber deposition at the wound site [141].

#### 6.2.3. Extracellular Vesicles Exhibit Anti-Inflammatory Effects

Mesenchymal stem cells naturally release EVs containing mitochondria, which can help rescue damaged cells. Yao et al. used metformin to stimulate adipose-derived stem cells to produce more mitochondria-containing EVs (Met-EVs). After testing them in radiation-damaged cells and tissues, they found that Met-EVs could restore mitochondrial function, reduce oxidative stress, and transfer active mitochondria to damaged cells. In addition, the construction of Met-EVs@Decellularized Adipose Matrix (DAM)/Hyaluronic Acid Methacrylic Acid (HAMA)-MNP contributes to a more stable release of Met-EVs. This Met-EVs@DAM/HAMA-MNP allows for better wound absorption of Met-EVs and promotes M2 macrophage polarization [142].

Platelet-rich plasma (PRP), PRP-derived exosomes (PRP-Exos), and MSC-derived exosomes (MSC-Exos) play a therapeutic role in wound healing. A study incorporated them into silk protein (SP)-based dual-crosslinked hydrogels, enhancing mechanical strength, prolonging GF release, and providing antimicrobial properties. These hydrogels exhibit anti-inflammatory effects by inhibiting NETosis, reducing oxidative stress, and modulating macrophages. In diabetic wounds, excessive NETs drive inflammation and tissue damage. Additionally, they shift macrophages from pro-inflammatory M1 (CD86) to anti-inflammatory M2 (CD206), promoting tissue regeneration and faster wound closure [143].

Flap transplantation has been widely used for treating wounds. However, ischemic necrosis at the distal end of the flap sometimes occurs. One study found that ABs from tissue cells adjacent to ischemic flaps can promote flap survival. These ABs can reduce ROS, promote endothelial cells and macrophages, shift macrophages from the M1 to the M2 phenotype, and inhibit ferroptosis. This regulation might be due to the miR-339-5p/KEAP1 axis. It offers a promising therapeutic approach to enhance flap survival in the wound healing process [144].

One clinical study investigated the application of adipose-derived stem cell (ASC)-conditioned medium and its derived exosomes (ASCE) in the treatment and repair of human acne scars. A randomization method was used to assign treatments to each half-face of the participants, with one side receiving exosome gel and the other receiving a control gel, both in combination with fractional CO_2_ laser treatment. The final follow-up revealed that the ECCA (a scoring system to evaluate the severity of acne scars) score on the exosome-treated side was significantly lower than that on the control side. Three-dimensional imaging showed significant reductions in atrophic scar volume, average pore volume, and skin surface roughness on the exosome-treated side. The therapeutic mechanisms may include anti-inflammatory effects, promotion of wound healing, collagen remodeling, and skin barrier restoration [145].

The derivation of EVs and their therapeutic effects in wound healing are summarized in Table 4.

## 7. Challenges for Clinical Application of Extracellular Vesicles and Future Direction

Despite the widespread use of EVs in the research and treatment of various diseases, the use of EVs still faces challenges.

### 7.1. Production Efficiency and Purity

A major challenge is the large-scale production of EVs. Using EVs in clinical therapy needs a large scale of production and high purity. However, EVs are naturally secreted by cells in small quantities. Currently, the isolation methods of EVs include differential ultracentrifugation, density-gradient ultracentrifugation, size-exclusion chromatography, precipitation kits, and immunoaffinity capture. Each method has its own efficiencies and limitations, which can affect overall production quality and purity. Producing enough EVs for therapy is currently inefficient and costly.

There are some approaches to enhance EV production. Changing the platform for larger-scale cell culture is one method, such as hyperflasks, hollow-fiber bioreactors, spinner bioreactors, and vertical wheels [65]. For example, hollow-fiber bioreactors are used to culture mesenchymal stem cells, from which EVs are then isolated on a large scale. Hollow-fiber bioreactors produced more MSCs compared with traditional cell culture and did not significantly affect the viability and differentiation capacity of MSCs [146].

In addition, existing EV production mainly relies on two-dimensional (2D) static cell culture. In a 2D environment, cells lack in vivo spatial polarization and architecture, which reduces cell–cell and cell–matrix interactions, leading to a decrease in the production of EVs [147]. Constructing three-dimensional tissues and giving them flow and stretching stimuli can significantly increase the production of EVs [148].

EV secretion is highly dependent on environmental conditions. Therefore, EV production per cell can also be increased by stimulating the cells with physical and chemical stimuli such as serum starvation, hypoxia, low pH, heat shock, and ultrasound [65,149].

To improve purity and yield, a combination of isolation methods can be conducted. For example, EVs from bone marrow-derived MSCs can be produced on a large scale when combining cross-flow filtration and ultracentrifugation. Cross-flow filtration can effectively reduce the initial volume, and ultracentrifugation is then used to efficiently concentrate and purify the EVs, ensuring high recovery and purity [146].

These methods for increasing EV production provide a reference for the future large-scale generation of EVs for clinical use. The scale of clinical application still needs to be further verified and improved.

### 7.2. Quality Control and Standardization

EVs can be highly heterogeneous in size, cargo, and functions, making their production more complicated [14]. The methods used to isolate and characterize EVs in different studies are diverse and lack standardization, resulting in poor data reproducibility and comparability.

Quality control during EV separation and characterization is important. For example, to enhance research reliability and reduce false positives, RNase, DNase, and protease protection assays can be performed. These assays involve treating EV samples with nucleases and proteases to assess whether their cargo is protected by the EV membrane. Resistance to enzymatic degradation indicates that the contents are truly encapsulated within the EVs. This approach significantly improves the accuracy and reproducibility of findings by ensuring that identified biomarkers or therapeutic agents are EV-associated rather than contaminants. However, many studies lack such rigorous validation, potentially compromising result accuracy [150].

The isolation process of EVs involves many variables. Batch-to-batch variability constitutes a significant obstacle to their application. Variables like cell type, growth conditions, bioreactor type, and media composition affect the final product. A better understanding of environmental factors influencing EV release can improve batch consistency [151].

The International Society for Extracellular Vesicles (ISEV) released the first Minimal Information for Studies of Extracellular Vesicles (MISEV) guidelines in 2014 [41], with subsequent updates addressing EV origin, isolation methods, quality control, molecular content, and characterization [24,152].

Adherence to MISEV enhances rigor and standardization in EV research, providing more reliable and stable results for clinical research and applications.

### 7.3. Safety Concerns

In clinical applications, EVs offer various advantages as potential therapeutic tools. However, they may also pose safety concerns.

When the source of EVs is allogenic or xenogenic, they may be recognized as foreign substances, triggering immune responses. Multiple factors, including source, size, surface composition, cargo, production and storage conditions, dosage, infusion rate, and biomolecular corona-influenced EV immunogenicity. For example, stem cell-derived EVs typically exhibit low immunogenicity, whereas tumor-derived EVs may possess immune-evasive properties. Gene editing can reduce MHC-I expression, thereby decreasing the immunogenicity of EVs [153].

Tumor-derived EVs may carry immune evasion factors, such as immunosuppressive molecules, which can impair the immune system’s ability to recognize and attack tumor cells [154]. This makes recipient cells more susceptible to tumor development or accelerates the growth of existing tumors.

The combination of EVs and viruses represents a highly promising therapeutic strategy. For instance, EVs can be an efficient system for delivering oncolytic adenoviral vaccines, significantly enhancing anti-melanoma efficacy and immune cell infiltration [66]. However, the use of viruses raises safety concerns, including the potential for residual viral replication, the risk of insertional mutagenesis, and uncertainties regarding long-term safety.

In summary, the safety of EVs should be carefully considered during their application. Prior to clinical use, their safety must be rigorously and repeatedly validated.

## 8. Conclusions and Outlook

Extracellular vesicles, including exosomes, microvesicles, and apoptotic bodies, have been widely investigated in recent years due to their pivotal roles in a wide range of diseases. These vesicles, released by various cell types, carry a cargo of proteins, lipids, and nucleic acids that influence cellular communication and can impact both normal physiological processes and disease progression.

In this review, we provided a comprehensive introduction to the structure and function of EVs, compared the properties of different types of EVs, and discussed various techniques for their isolation, identification, and manufacturing processes.

We focused on the role of EVs in four major skin conditions: psoriasis, atopic dermatitis, systemic lupus erythematosus, and wound healing. In these conditions, EVs exhibit a complex dual role, functioning both as contributors to disease pathology and as potential therapeutic tools.

Regarding pathology, EVs play a critical role in immune modulation, serving as mediators of cell-to-cell communication that can either promote or regulate immune responses.

Regarding therapy, EVs can be non-invasive biomarkers for diagnosis and monitoring processes. Moreover, both natural and engineered EVs are being explored as therapeutic agents.

Research on EVs still faces several challenges, particularly regarding their clinical application. Key issues include low production efficiency and purity, lack of quality control and standardization, and safety concerns. Future studies may explore strategies to enhance EV yield, such as scaling production, employing 3D cell culture systems, or stimulating donor cells. The combination of different EV isolation and enrichment techniques may further improve purity. Standardization should also be addressed during the isolation process; adherence to the MISEV guidelines can enhance experimental rigor and ensure reproducibility. Moreover, EVs’ immunogenicity and overall safety profile must be thoroughly evaluated before clinical translation.

In conclusion, emerging therapeutic approaches based on EVs may provide innovative and promising treatments for skin diseases.

## Figures and Tables

**Table 1:** Summary of the derivation and therapeutic effects of extracellular vesicles (EVs) in psoriasis.

Diseases	EVs Derived From	Function	Reference
Psoriasis	Exosomes derived from autologous adipose-derived MSCs	↓ Skin thickness, erythema, and inflammatory markers (IL-23, TNFα, IL-17, and IFNγ) ↑ Anti-inflammatory factor IL-10 and FOXP3	[94]
Psoriasis	IFNγ-sEVs derived from HUCMSCs	↓ Immune cell proliferation and activation ↓ Psoriasis symptoms by modulating Th17/Th2 balance ↓ Inflammatory cytokines ↑ Delivery of ASO-210 (which alleviates psoriasis)	[95]
Psoriasis	EV derived from umbilical cord blood mononuclear cell	↑ Macrophages to an anti-inflammatory phenotype ↓ CD4^+^ and CD8^+^ T cell proliferation and inflammatory markers ↑ Treg	[96]
Psoriasis	Gut microbiota-derived OMVs from *Parabacteroides goldsteinii*	↓ Psoriasis-like skin inflammation by targeting affected areas On macrophage: ↓ ROS level and M1 phenotype macrophage polarization On T cells: ↓ Differentiation of CD4^+^ T cells into Th1 and Th17 cells On DCs: ↓ Maturation of LPS-induced BMDCs and inflammatory cytokine On keratinocytes: ↓ Proliferation rate	[97]
Psoriasis	EVs from *Cutibacterium acnes*, encapsulated in a sustained-release hydrogel (CA-EVs@GHM)	↓ Psoriasis symptoms Restore skin microbiota balance: ↓ Staphylococcus colonization, ↑ microbial diversity ↓ Conversion of ILC2 to pathological ILC3	[98]
Psoriasis	PD-L1^+^ exosomes derived from melanoma cells carrying pristimerin (anti-inflammatory compound)	↓ Psoriasis by targeting CD4^+^ T cells and keratinocytes. ↓ Th17 proliferation ↑ Treg differentiation ↓ Inflammation and ferroptosis-related changes	[99]

↑: Upregulation, activation, or increased expression, activity, or level; ↓: Downregulation, suppression, or decreased expression, activity, or level. MSCs: Mesenchymal stem cells; IFNγ-sEVs: Small extracellular vesicles stimulated by interferon-γ; HUCMSCs: Human umbilical cord mesenchymal stem cells; OMVs: Outer membrane vesicles; ROS: Reactive oxygen species.

**Table 2:** Summary of the derivation and therapeutic effects of extracellular vesicles (EVs) in atopic dermatitis (AD).

Diseases	EVs Derived From	Function	Reference
AD	Nanoencapsulated EVs derived from canine MSCs	↓ Inflammatory cytokine expression (IL-2, IL-4, IL-5, IL-6, IL-10, IL-13, and IL-31) in human keratinocytes Nanoencapsulation with calcium alginate: ↑ EV delivery, stability, and therapeutic efficacy	[116]
AD	EVs derived from MSC overexpressing extracellular SOD3	Deliver SOD3 protein, improving therapeutic efficacy: ↓ AD symptoms by modulating immune cell activation and differentiation	[117]
AD	EVs derived from *Lactobacillus plantarum*	↓ IL-6 secretion in keratinocytes and macrophages ↑ Cell viability ↓ SA-EV-induced skin inflammation ↓ Epidermal thickness ↓ IL-4 levels	[118]
AD	EVs derived from *Limosilactobacillus fermentum* SLAM216	↓ Immunoregulatory cytokines ↓ Epidermal thickness and mast cell infiltration ↑ Skin barrier function ↑ Serotonin synthesis ↓ Scratching and depression-related behaviors	[119]
AD	EVs derived from *Pinctada martensii* mucus	↓ Inflammation by inhibiting the FOXO3/NLRP3 signaling pathway miR-100-5p in EVs involved in anti-inflammatory effects	[120]

↑: Upregulation, activation, or increased expression, activity, or level; ↓: Downregulation, suppression, or decreased expression, activity, or level. MSCs: Mesenchymal stem cells; SOD3: Superoxide dismutase 3; SA-EVs: *Staphylococcus aureus*-derived extracellular vesicles.

**Table 3:** Summary of the derivation and therapeutic effects of extracellular vesicles (EVs) in systemic lupus erythematosus (SLE).

Diseases	EVs Derived From	Function	Reference
SLE	Exosomes derived from bone marrow mesenchymal stem cells	↑ Anti-inflammatory polarization of macrophages (↑ CD206, B7H4, CD138, Arg-1, CCL20, and anti-inflammatory cytokines secretion) ↓ T cell infiltration in the kidney ↑ IL-17^+^ Tregs ↑ Macrophage efferocytosis	[130]
SLE	Exosomes derived from MSCs containing tsRNA-21109	↓ Macrophage M1 polarization markers (CD80, NOS2, MCP-1) ↑ Macrophage M2 polarization markers (CD206, ARG1, MRC-2) ↓ TNF-α and IL-1β	[131]
SLE	Exosomes derived from UC-BSCs	↓ Th17 cell differentiation ↑ Treg cells ↓ Pro-inflammatory cytokines (TNF-α, IL-6, and IL-17) ↑ Anti-inflammatory cytokines (IL-10 and TGF-β)	[132]
SLE	Apoptotic vesicles derived from BMMSCs	↓ Lymphoproliferation ↓ IFN-γ^+^ CD4^+^ T cells ↑ Foxp3^+^ regulatory T cells ↓ T cell activation (via direct contact, independent of macrophages) ↓ Th1 and Th17 cells	[133]
SLE	EVs derived from stem cells of human exfoliated deciduous teeth	↓ SLE-like symptoms ↑ Hematopoietic niche formation and immunoregulation	[134]

↑: Upregulation, activation, or increased expression, activity, or level; ↓: Downregulation, suppression, or decreased expression, activity, or level. BMMSCs: Bone marrow mesenchymal stem cells; MSCs: Mesenchymal stem cells; UC-BSCs: Umbilical cord blood mesenchymal stem cells.

**Table 4:** Summary of the derivation and therapeutic effects of extracellular vesicles (EVs) in wound healing.

Diseases	EVs Derived From	Function	Reference
Wound Healing	Exosomes derived from ASC (ASC-EXOs)	↑ Dermal fibroblast proliferation, migration, and collagen production ↑ Wound healing and tissue regeneration Combination of ASC-EXOs and hyaluronic acid: ↑ Wound closure rates and tissue remodeling	[136]
Wound Healing	EVs derived from macrophage	↑ Wound healing in diabetic obese mice ↑ Expression of adiponectin (insulin-sensitizing properties) ↑ Intercellular signaling, ↓ inflammation, ↑ wound closure ↑ Dermal fibroblast proliferation and basal keratinocyte activation	[137]
Wound Healing	EVs derived from platelets	↑ Dermal fibroblast proliferation, migration, and angiogenesis	[138]
Wound Healing	EVs derived from serum- and glucose-deprived HUCMSCs	↑ Angiogenesis and skin wound healing ↑ Migration, proliferation, and tube formation of endothelial cells ↑ VEGFA production (contributes to tissue regeneration) Pathway: ↑ miR-29a-3p, ↓ CTNNBIP1, and ↑ Wnt/β-catenin signaling pathway	[139]
Wound Healing	EVs derived from MSC combined with the HDAC7-derived peptide 7A in a hydrogel	Anti-inflammatory, pro-angiogenic, and pro-proliferative effects ↑ Diabetic wound healing ↑ Fibroblast migration and proliferation ↑ Anti-inflammatory macrophages ↓ NF-κB signaling pathway; ↑ TGF-β expression	[140]
Wound Healing	EVs derived from epidermal stem cells containing VH298 (a HIF-1α stabilizer)	↑ Angiogenesis-related protein (HIF-1α and VEGFA) ↑ Endothelial cell function Commination with GelMA hydrogel: ↑ Wound healing via↑ local blood supply and HIF-1α/VEGFA signaling	[141]
Wound Healing	Mitochondria-rich EVs from metformin-treated stem cells	↑ Mitochondrial function: Restoring membrane potential, ↑ ATP levels, ↓ oxidative stress Combination with hydrogel microneedle patch: ↑ M2 macrophage polarization and healing in radiation-induced chronic wounds	[142]
Wound Healing	Exosomes derived from platelet-rich plasma and MSCs combined with silk protein hydrogel	Hydrogels enable sustained release of growth factors and exosomes ↓ Matrix metalloproteinase-9 expression ↑ Anti-NETotic effect, angiogenesis, and re-epithelialization	[143]
Wound Healing	ABs derived from fibroblast-like cell	↑ Ischemic flap survival ↓ Ferroptosis and oxidative stress in endothelial cells and macrophages ↑ M2 macrophage polarization through the miR-339-5p/KEAP1/Nrf2 axis	[144]
Wound Healing	Exosomes derived from ASC	↓ The ECCA scores (a tool for evaluating the severity of atrophic acne scars) of acne patients ↓ Volume of atrophic scars, mean volume of skin pores, and skin surface roughness	[145]

↑: Upregulation, activation, or increased expression, activity, or level; ↓: Downregulation, suppression, or decreased expression, activity, or level. ASC: Adipose-derived stem cell; MSCs: Mesenchymal stem cells; HUCMSCs: Human umbilical cord mesenchymal stem cells; HDAC7: Histone deacetylase 7; 7A: 7-amino-acid peptide; HIF-1α: Hypoxia-inducible factor 1 alpha; GelMA: Gelatin methacryloyl; NET: Neutrophil extracellular trap; KEAP1/Nrf2 axis: Kelch-like ECH-associated protein 1-NF-E2-related factor 2 axis.

## Abbreviations

The following abbreviations are used in this manuscript:

ABs	Apoptotic bodies
AD	Atopic dermatitis
ApoEVs	Apoptotic extracellular vesicles
BSA	Body surface area
DGUC	Density gradient ultracentrifugation
DUC	Differential ultracentrifugation
ECs	Endothelial cells
EPCs	Endothelial progenitor cells
EVs	Extracellular vesicles
FLG	Filaggrin
HA	Hyaluronic acid
HCs	Healthy controls
HUVECs	Human umbilical vein endothelial cells
IC	Immune complex
LN	Lupus nephritis
MalaEx	Malassezia releases extracellular vesicles
MMPs	Matrix metalloproteinases
MSC	Mesenchymal stem cells
MVBs	Multivesicular bodies
MVs	Microvesicles
NET	Neutrophil extracellular traps
NETosis	Neutrophil extracellular traps formation
NTA	Nanoparticle tracking analysis
PASI	Psoriasis area and severity index
PHKs	Primary human keratinocytes
PsA	Psoriatic arthritis
*S. aureus*	*Staphylococcus aureus*
SEC	Size-exclusion chromatography
sEVs	Small extracellular vesicles
SLE	Systemic lupus erythematosus
TEM	Transmission electron microscopy
TFF	Tangential flow filtration
Treg	Regulatory T cells
UCB	Umbilical cord blood
PRP	Platelet-rich plasma
